# Development and Validation of Artificial Intelligence Addiction Scale for Researchers: A Methodological Study

**DOI:** 10.1155/jonm/8458533

**Published:** 2025-12-18

**Authors:** Ahmed Abdelwahab Ibrahim El-Sayed, Samira Ahmed Alsenany, Maha Gamal Ramadan Asal, Ibrahim Alasqah

**Affiliations:** ^1^ Nursing Administration Department, Faculty of Nursing, Alexandria University, Alexandria, Egypt, alexu.edu.eg; ^2^ Public Health Department, Faculty of Nursing, King Abdulaziz University, Jeddah, Saudi Arabia, kau.edu.sa; ^3^ Medical Surgical Nursing Department, Faculty of Nursing, Alexandria University, Alexandria, Egypt, alexu.edu.eg; ^4^ Department of Community, Psychiatric, and Mental Health Nursing, College of Nursing, Qassim University, Buraydah, 51452, Saudi Arabia, qu.edu.sa

**Keywords:** addiction, artificial intelligence, methodological study, psychometric, researchers, scale, validation

## Abstract

**Background:**

The integration of artificial intelligence (AI) tools into research has brought significant advancements, enhancing efficiency, innovation, and productivity across various academic disciplines. However, alongside these transformative benefits, the growing dependence on AI tools has raised concerns regarding overreliance and the potential for addictive behaviors among researchers. Despite the widespread adoption of AI among the researchers, there remains a notable gap in the availability of validated instruments specifically designed to assess AI addiction within this context.

**Objective:**

To develop a scale to measure AI addiction among researchers and evaluate its psychometric properties.

**Design:**

A methodological design was employed, consisting of two phases: scale development and psychometric evaluation.

**Methods:**

Items were generated through a comprehensive literature review and semistructured interviews to capture AI addiction attributes. The scale’s psychometric properties—including content validity, face validity, construct validity, and internal consistency reliability—were assessed. Data from a convenience sample of 718 nursing researchers were randomly divided into two independent subsamples for exploratory factor analysis (EFA) and confirmatory factor analysis (CFA). Reliability was evaluated using Cronbach’s alpha, McDonald’s omega, split‐half reliability, and corrected item–total correlations.

**Results:**

The finalized scale comprises 22 items across five dimensions: compulsive behavior, overdependency, functional impairment, withdrawal, and tolerance. EFA identified a five‐factor structure explaining 73.66% of the variance. CFA validated the structure with robust fit indices for first order (*χ* ^2^/DF = 2.289, CFI = 0.962, and RMSEA = 0.06) and second order (*χ* ^2^/DF = 2.243, CFI = 0.962, and RMSEA = 0.059) models. The scale demonstrated excellent internal consistency and reliability, with Cronbach’s alpha (*α* = 0.924), McDonald’s omega (*ω* = 0.870), and a Spearman–Brown split‐half coefficient of 0.814. Moderate interfactor correlations (*r* = 0.41–0.62) confirmed its multidimensionality.

**Conclusion:**

The researchers’ AI addiction scale is a valid and reliable tool for assessing AI addiction among researchers, providing a robust framework to evaluate compulsive behavior, dependency, functional disruption, withdrawal symptoms, and tolerance.

## 1. Introduction

Artificial intelligence (AI) has rapidly transformed research practices across disciplines. Tools such as ChatGPT, Gemini, GitHub Copilot, and Poe have emerged as integral to the academic workflow, streamlining tasks such as literature synthesis, data analysis, manuscript drafting, and hypothesis generation [1, 2]. These AI‐driven platforms, underpinned by sophisticated language models, are lauded for enhancing research efficiency and productivity, offering instant access to high‐quality outputs [3].

Beyond these core functions, AI is increasingly used for multimodal data processing, language translation, automated summarization, and advanced statistical modeling—conveniences that reduce research time and expand opportunities for international collaboration [4, 5]. Collectively, these tools have reshaped the contours of scholarly work by accelerating information retrieval, democratizing access to sophisticated analytic techniques, and facilitating cross‐border academic exchange in unprecedented ways [3].

At the same time, researchers face serious risks associated with this accelerated adoption. While AI tools promise convenience, they also invite challenges such as plagiarism, diminished originality, data bias, breaches of privacy, overstandardization of academic writing, and unresolved ethical questions about transparency and accountability [6–8]. Some risks are structural, arising from the limitations of training data and the opacity of algorithms; others are behavioral, emerging from the ways in which scholars themselves interact with AI. The convergence of these structural and behavioral risks underscores a central paradox: AI has the potential to be both a catalyst for intellectual innovation and a vehicle for scholarly erosion [9].

This growing reliance on AI tools raises important questions about cognitive independence and professional integrity [10]. In the context of research—where intellectual rigor, evidence‐based reasoning, and methodological precision are indispensable—the frequent outsourcing of cognitive tasks to AI may gradually erode critical scholarly competencies [11]. Activities once performed through reflective thought, theoretical synthesis, and iterative writing are now often delegated to AI‐driven systems. While the efficiency gains are clear, this shift also blurs the boundary between legitimate academic support and the abdication of intellectual responsibility. Scholars have warned that such trends signal a tipping point: when AI transitions from being an enabling instrument to functioning as a cognitive crutch [12]. At this juncture, the risk transcends technological dependency and begins to resemble behavioral addiction, defined by compulsive use, psychological reliance, and functional impairment [13].

Although the notion of AI addiction is still contested, its conceptualization can be meaningfully anchored within established theoretical traditions on behavioral addiction. Griffiths’ [14] components model of addiction, which delineates salience, mood modification, tolerance, withdrawal, conflict, and relapse, has been applied across a range of nonsubstance‐related behaviors, from gambling to internet use. It offers a comprehensive framework for identifying when high engagement crosses into problematic use. In parallel, the DSM‐5 criteria for substance use disorders emphasize psychological preoccupation, progressive escalation of use, and clinically significant impairment in social or occupational functioning—criteria that increasingly mirror patterns observed in technology‐mediated behaviors [15, 16]. Together, these frameworks provide both conceptual clarity and empirical grounding for analyzing problematic AI engagement in academic contexts.

The present study draws explicitly on these theoretical foundations. Each of the five dimensions identified—compulsive behavior, overdependency, functional impairment, withdrawal, and tolerance—maps onto constructs from Griffiths’ model and DSM‐5. Compulsive behavior reflects the salience of AI use, where engagement becomes automatic, irresistible, and often detached from conscious deliberation. Overdependency is conceptually linked to the conflict dimension, capturing the way in which excessive reliance disrupts autonomy and undermines self‐efficacy. Functional impairment corresponds to DSM‐5 criteria describing disruptions in professional performance and academic productivity.

Withdrawal encompasses the emotional and cognitive distress experienced when AI tools are inaccessible, echoing both DSM‐5 withdrawal symptoms and Griffiths’ characterization of negative affect in the absence of use. Finally, tolerance represents the escalating trajectory of use, where increasing intensity or frequency of engagement is needed to obtain the same cognitive or scholarly outcomes. These five dimensions, therefore, are not arbitrarily chosen but emerge directly from robust theoretical and clinical traditions [12, 17, 18].

Importantly, leading scholars caution against hastily pathologizing everyday digital behaviors. Aarseth et al. [19]; Kardefelt‐Winther et al. [20]; and Billieux et al. [17] highlighted the danger of medicalizing normal variations in technology use without sufficient contextual nuance. Excessive use does not necessarily equal addiction, and high engagement can coexist with positive outcomes. For this reason, the present study does not aim to label frequent AI use as inherently harmful. Rather, it seeks to identify the conditions under which use shifts from beneficial integration to compulsive engagement and functional impairment.

Compulsive behavior is a core feature of behavioral addiction and is increasingly observed among researchers who reflexively turn to AI tools for even the most routine academic tasks [5, 21]. This compulsive engagement is often rationalized as productive, yet it bypasses vital cognitive processes such as hypothesis formation, synthesis, and critical review—replacing intellectual labor with automated outputs [22]. In research environments where speed and volume are often prioritized, such compulsivity may be normalized, further reinforcing reliance on AI [23].

Closely linked is the dimension of overdependency, defined by the emotional and psychological belief that one cannot function effectively without AI. This dependency is distinct from high engagement and reflects a shift in academic self‐efficacy and cognitive autonomy [11, 12]. Over time, researchers may begin to doubt their capacity to analyze, write, or critique without AI input—developing a form of learned helplessness that undermines their professional identity and intellectual agency [24]. This is particularly concerning for early‐career scholars, who are still consolidating methodological expertise and theoretical fluency. For them, dependence on AI may obstruct the development of essential scholarly competencies. While these dynamics are especially pressing in disciplines where evidence integrity carries direct social or clinical implications—such as nursing, medicine, and education—they generalize across fields where originality and cognitive autonomy remain the cornerstones of academic contribution [25].

Functional impairment, another hallmark of behavioral addiction, refers to the negative impact of excessive AI use on academic performance and research quality [22]. Studies have shown that reliance on digital tools can lead to superficial analyses, diminished methodological rigor, and reduced originality [8, 26]. These impairments have practical consequences: for some fields, they weaken the evidentiary base for policy or practice; for others, they diminish theoretical contributions and curtail innovation. Ultimately, functional impairment threatens the scholarly mission itself, which depends not merely on producing output but on advancing knowledge through originality and depth [6]. More broadly, such impairments may result in fragmented argumentation, a homogenization of research outputs, and diminished long‐term scientific progress [9].

Withdrawal symptoms—such as anxiety, frustration, or helplessness when AI tools are unavailable—are increasingly reported by researchers, particularly during internet outages or institutional restrictions [18, 27]. This distress reflects psychological entrenchment and echoes findings from earlier research on internet and smartphone withdrawal [28]. In high‐stakes academic settings, the inability to access AI tools can disrupt workflow and reduce productivity, exposing a level of dependency that transcends convenience [7].

Equally concerning is tolerance, defined as the progressive escalation of use. Initial reliance may be limited to grammar corrections or text summarization, but over time, researchers may extend AI use to conceptual ideation, analytical interpretation, and even drafting peer review responses. This intensification mirrors the tolerance patterns described in Griffiths’ model and DSM‐5 [24, 29]. As tolerance develops, researchers often struggle to produce work without AI, entrenching a cycle of reliance that further blurs the boundary between augmentation and substitution [30]. Recent empirical studies confirm that this escalation is not hypothetical but measurable, reflecting identifiable behavioral trajectories [31].

Despite growing recognition of these risks, existing instruments are ill‐equipped to measure AI‐specific dependencies in academic contexts. Tools such as the internet Addiction Test [21], the Smartphone Addiction Scale [32], and the Generalized Problematic Internet Use Scale [33] were designed for recreational or generalized digital use and thus fail to capture the unique demands of scholarly engagement. Even the recently developed AI‐Dependence Scale [12], while valuable, was designed primarily for students and does not fully reflect the cognitive, professional, and psychological challenges faced by active researchers. Scholars further caution that applying generalized digital addiction measures to academic contexts without theoretical adaptation risks both overpathologizing normal behaviors and overlooking context‐specific vulnerabilities [10, 15, 34].

The context of AI use in academic research is therefore uniquely complex. Unlike recreational technologies, academic AI use is goal‐directed, cognitively demanding, and professionally consequential. As such, it requires instruments sensitive to the fine line between intensive engagement and addiction. Scholars have urged that behavioral addiction measures must be tailored to context, making clear distinctions between high engagement and pathological involvement [17, 35, 36]. Without such nuance, there is a risk of both underdiagnosing genuinely harmful patterns and overpathologizing normative behavior.

In light of these concerns, the development of a new instrument capable of capturing the cognitive, emotional, and behavioral dimensions of AI overuse in research is both urgent and necessary. The present study addresses this gap by developing and validating the Researcher Artificial Intelligence Addiction Scale (RAIAS). To maximize the scale’s applicability and policy relevance, we designed the instrument to target researchers broadly rather than a single discipline. The five dimensions of the RAIAS are grounded in theoretical models of behavioral addiction (Griffiths’ component model and DSM‐5 criteria) that are domain‐agnostic; these constructs capture psychological and behavioral processes common to problematic technology use across contexts [14, 15]. The RAIAS items were therefore written to assess universal manifestations of problematic AI engagement, rather than discipline‐specific tasks. This conceptual design supports broader relevance while remaining sensitive to the research environment.

## 2. Methods

### 2.1. Research Design

The purpose of this methodological study was to develop a two‐phase RAIAS, designed as an assessment tool to measure the frequency of AI addiction behaviors among researchers. The development process involved two main phases: in the first phase, a comprehensive literature review was conducted to identify relevant dimensions and items of AI addiction, followed by qualitative interviews and systematic item generation. In the second phase, the psychometric properties of the constructed scale were evaluated.

### 2.2. Phase 1: Scale Development

#### 2.2.1. Domain Identification and Items Generation

##### 2.2.1.1. Literature Review

To build the conceptual foundation, a comprehensive literature review was conducted, focusing on digital addiction behaviors within academic and professional contexts. Systematic searches were executed across major academic databases, including CINAHL, PubMed, and Google Scholar, using specific terms like “digital addiction,” “AI addiction,” “AI usage,” and “psychometric validation.” Inclusion was limited to peer‐reviewed articles published between 2015 and 2025 that addressed AI or digital addiction behaviors in academic settings, while nonrelevant studies or those lacking discussion on behavioral or psychological aspects were excluded. This systematic review yielded 37 articles, with 11 deemed directly relevant, which informed the identification of the scale’s core components. From these sources, 23 items were generated, representing the five core dimensions of AI addiction that would structure the RAIAS: compulsive behavior, overdependency, functional impairment, withdrawal, and tolerance.

##### 2.2.1.2. Qualitative Data Collection

To capture the real‐world behaviors and attitudes specific to researchers’ use of AI, the literature findings were complemented by semistructured interviews. Seven researchers (ages 32–46, 4 males, 3 females, with varying levels of experience in AI use, from 1 to 4 years) using AI tools in their work participated. These interviews, lasting 15 to 20 min each, explored detailed aspects of AI usage, including time spent on AI tasks, emotional responses, and the perceived impacts on productivity.

The participants reported using AI tools in various aspects of their research work, such as data analysis, literature review, and paraphrasing. They indicated spending an average of 3 h per day using AI, with some using it more intensively during specific project phases like data processing or writing papers. Emotional responses to AI use were mixed, while some participants expressed frustration with its limitations, others highlighted its positive impact on productivity and efficiency. Many participants noted a sense of dependency, as they increasingly relied on AI for routine tasks, and some felt uncomfortable or unsure about their research outputs when AI tools were unavailable. Despite these concerns, most participants acknowledged that AI had a generally positive effect on productivity, allowing them to complete tasks more quickly. However, a few expressed worries about losing critical thinking and creativity in their work due to overreliance on AI.

Following informed consent and confidentiality briefings, the interview transcripts were analyzed using a combined inductive and deductive approach [37]. Open coding identified meaningful phrases related to AI usage, which were then grouped into categories and mapped to the five addiction dimensions identified in the literature. A second researcher independently reviewed the coding for consistency, and participants provided feedback on anonymized excerpts to enhance the validity and minimize researcher bias. This qualitative process successfully generated 9 additional items, ensuring the scale’s relevance to the lived experiences of the target population.

##### 2.2.1.3. Item Pool Refinement and Scaling

The items generated from the literature and those from the interviews were then merged. After careful review, two overlapping items were removed, resulting in an initial pool of 30 items. This entire pool was then subjected to a collaborative review by two researchers who scrutinized each item for clarity, relevance, and alignment with the five defined addiction dimensions. Ambiguous or double‐barreled items (those addressing more than one concept) were removed during this rigorous process, yielding a final preliminary set of 28 items for content validity assessment (see Supporting). Adhering to DeVellis and Thorpe [38] guidelines for robust scale construction, each item was carefully worded to address a single, distinct concept. Each item is rated on a 5‐point Likert scale ranging from 1 (rarely) to 5 (always), with higher scores indicating a greater frequency of behaviors associated with problematic AI use.

#### 2.2.2. Content Validity

After generating the preliminary items, we conducted a content validity assessment to ensure that the items accurately reflected the attributes of researchers’ AI addiction.

The experts reviewed the items to assess their relevance and clarity. The experts rated the relevance of the items via a four‐point scale: 1 for “not relevant,” 2 for “somewhat relevant,” 3 for “quite relevant,” and 4 for “highly relevant.” A binary scoring system was employed, where the first two responses (1 and 2) were assigned a score of 0, and the latter two (3 and 4) were scored as 1. Content validity was assessed via the item content validity index (I‐CVI) for each item and the scale content validity index (S‐CVI) for the overall scale. According to [39], an I‐CVI score greater than 0.79 is considered acceptable. Items with I‐CVI scores between 0.70 and 0.79 require revision, whereas those scoring below 0.70 should be eliminated. For the S‐CVI, an overall value greater than 0.90 is necessary to retain the scale. In addition, the experts were asked to indicate how to improve the content if applicable for each item.

#### 2.2.3. Pilot Testing for Face Validity

To assess face validity, a pilot test was conducted with 40 researchers from Alexandria University, Egypt, who actively used AI tools in their research. This sample size meets the minimum recommendation for pilot studies in scale development, as noted by Johanson and Brooks [40], who suggest at least 30 participants. The inclusion criteria for participation required that researchers hold a master’s or doctoral degree in nursing, were actively engaged in research, and reported using AI tools such as ChatGPT, Gemini, GitHub Copilot, or Poe for tasks like literature review, data analysis, or manuscript preparation at least three times per week over the past 3 months. Additionally, participants needed to have at least 1 year of independent research experience and provide informed consent before participating. 52 researchers were screened to ensure they met these criteria. Of those screened, 9 were excluded for not meeting the criteria and 3 declined to participate, leaving a final sample of 40 participants. To assess the readability, clarity, and relevance of the scale items, participants were asked to rate each item using a 5‐point Likert scale. All completed questionnaires were collected in sealed envelopes. These pilot participants were not included in the main psychometric evaluation phase to prevent bias in subsequent analyses.

### 2.3. Phase 2: Psychometric Evaluation

#### 2.3.1. Sample and Data Collection

The second phase of the study aimed to evaluate the psychometric properties of the RAIAS. This phase sought to empirically validate the scale among a diverse sample of nursing researchers from Egypt and Saudi Arabia. The sample size for this phase was determined on recommendations by Guadagnoli and Velicer [41], who suggested a minimum of 10 participants per scale item, and by MacCallum et al. [42], who recommended at least 300 participants for each factor analysis; accordingly, a target sample of at least 600 researchers was set.

The data collection utilized an online survey administered via Google Forms and was open to participants from March to June 2024. Prior to accessing the questionnaire, respondents were presented with an information sheet detailing the study’s purpose, voluntary nature, and expected duration. This sheet explicitly emphasized the strict maintenance of anonymity and confidentiality, guaranteeing that no personally identifying information would be collected, and encouraged honest responses by stating there were no right or wrong answers. Access to the survey was strictly conditional upon providing electronic informed consent. The instrument was organized into three sections: Screening and Eligibility Verification (based on pilot study criteria), Demographic Characteristics (gathering background data such as age, qualification, and research experience), and the RAIAS Items.

Recruitment involved the direct distribution of approximately 1900 invitations via targeted professional social media platforms, including specialized Facebook and WhatsApp research groups. This direct outreach was intentionally supplemented by snowball sampling, where initial respondents were asked to share the link with other eligible researchers specifically within the nursing field and study setting. This dual approach yielded a total of 763 responses. Following screening, 43 responses were excluded for not meeting the established inclusion criteria, resulting in a final eligible sample of 720 participants. The effective response rate, calculated exclusively against the 1900 directly invited individuals, was 37.9%. This final sample size successfully exceeded the planned requirements for the study. For data security, all responses were stored in a secure, password‐protected folder accessible solely to the principal investigator.

#### 2.3.2. Ethical Considerations

The study protocol was approved by the Research Ethics Committee of the Faculty of Nursing at Alexandria University, Egypt (IRB00013620 AU:20‐8‐419). The study fully adhered to the ethical principles outlined in the Declaration of Helsinki. Before participating, all the respondents provided electronic informed consent, detailing the study’s purpose, voluntary nature, confidentiality of responses, and participants’ right to withdraw at any time.

#### 2.3.3. Statistical Analysis

For the final analysis, the data were processed via IBM SPSS Statistics, Version 28, and AMOS, Version 28. Initially, the data were screened for extreme outliers, resulting in the identification of two cases with extreme values, which were subsequently excluded from further analysis. The remaining 718 responses were then divided into two equal halves via the “random sample of cases” option, yielding two subsamples of 359 responses each. These subsamples were designated as follows: Subsample 1 for EFA and Subsample 2 for CFA. Before conducting factor analyses, the data for each subsample were examined for normality through the assessment of skewness and kurtosis values. Specifically, absolute values of kurtosis greater than 7 and skewness exceeding 3 were considered indicative of non‐normal distributions [43], guiding the choice of statistical tests for subsequent analyses.

Using the data from Subsample 1, construct validity was assessed using EFA employing principal axis factoring (PAF) with Promax rotation and Kaiser normalization to identify the underlying factor structure of the item pool. The PAF was chosen for its capacity to identify latent constructs by emphasizing the shared variance among observed variables, aligning with the objective of scale validation. This approach isolates common variance, providing a clearer understanding of the underlying factors that represent the constructs measured by the scale items, which enhances the accuracy of the scale’s dimensionality assessment [44]. Promax, an oblique rotation method, was used because it allows factors to correlate, which is appropriate when interrelated constructs are anticipated. This oblique rotation enhances interpretability by producing a more realistic factor structure for complex, related constructs, such as AI addiction behaviors [44].

The data’s suitability for EFA was evaluated via the Kaiser–Meyer–Olkin (KMO) measure and Bartlett’s test of sphericity. A KMO value above 0.70 is acceptable, and a significant Bartlett’s test (*p* < 0.05) confirms the correlation matrix’s appropriateness for factor analysis. Factors with an eigenvalue of ≥ 1.0 were extracted, and items with a factor loading of ≥ 0.50 were retained [45].

Following EFA, CFA was conducted to test the proposed factor structure identified in the EFA using Subsample 2. Model fit was assessed via multiple fit indices, with recommended cut‐off points employed to determine the adequacy of fit. A chi‐square to degree‐of‐freedom ratio (*χ* ^2^/DF) value less than 2 indicates good fit [46], and values less than 5 are considered acceptable [47]. The root mean square error of approximation (RMSEA) should ideally be ≤ 0.06 for a close fit; however, values up to 0.08 are often deemed acceptable [48, 49]. The goodness‐of‐fit index (GFI) should meet or exceed 0.90 for adequate model fit, although higher cut‐offs (≥ 0.95) are preferable in studies with low factor loadings or smaller sample sizes [50]. The root mean square residual (RMR) is also recommended to be ≤ 0.08 [49]. Furthermore, both the Tucker–Lewis’s index (TLI) and the comparative fit index (CFI) should exceed 0.90 to confirm good model fit, with the incremental fit index (IFI) also recommended to exceed 0.90 for strong model support [51].

In addition to measuring the model fit, the convergent validity, discriminant validity, and reliability were evaluated. Convergent validity was assessed via standardized factor loading, composite reliability, and average variance extracted (AVE); if their values were higher than 0.5, 0.70, and 0.50, respectively, the questionnaire was considered to have satisfactory convergent validity [52].

Discriminant validity was evaluated via the Fornell and Larcker [53] criterion, which compares the square root of the AVE for each factor to the interfactor correlations. The square root of the AVE for each factor exceeded the correlations with other factors, indicating satisfactory discriminant validity.

To assess the internal consistency and reliability of the scale, Cronbach’s alpha (*α*) and McDonald’s omega (*ω*) were calculated. Consistent with the guidelines proposed by George and Mallery [54], *α* values between 0.7 and 0.8 indicate acceptable reliability, 0.8 to 0.9 reflect good reliability, and coefficients above 0.9 demonstrate excellent reliability. For McDonald’s omega, a cut‐off point of *ω* ≥ 0.70 was applied to confirm acceptable reliability. Items were further evaluated using the corrected item–total correlation (C‐ITC); items were considered for elimination if their C‐ITC values fell below 0.3 and if their removal significantly increased the overall α [45]. In addition, split‐half reliability was calculated using the Spearman–Brown coefficient, with a minimum acceptable value set at 0.70.

## 3. Results

### 3.1. Phase 1: Preliminary Analysis of the Researcher AI Addiction Scale

#### 3.1.1. Content and Face Validity

A panel of 12 experts participated in the content validity assessment (7 in the first round and 5 in the second round), all holding doctoral degrees in relevant fields: four in psychology, four in digital health, three in nursing and health sciences, and one in behavioral addiction and mental health. The panel included eight full professors and four associate professors, with an average of 16.3 years of professional and research experience (range 12–23 years). Their ages ranged from 42 to 56 years.

In the first round, the S‐CVI was 0.908, and the I‐CVI scores ranged from 0.428 to 1.00. Four items (items # 9, 20, 23, 27) with I‐CVI scores below 0.60, which also had overlapping meanings based on the expert opinion, were deleted to enhance clarity and precision. In the second round, both the S‐CVI and I‐CVI met the predefined validity criteria, with an S‐CVI of 0.915 and all I‐CVI scores ≥ 0.8. As the scale had reached satisfactory validity, no further suggestions were made by the experts, and the item pool was finalized.

Following the expert assessment, the face validity of the scale was evaluated through a pilot study. The participants rated the readability and clarity of the remaining 24 items using a 5‐point Likert scale. The average readability score was 4.9 ± 0.378, and the average clarity score was 4.85 ± 0.426, indicating that there were no significant issues with the clarity or readability of the items. The average time required to complete the scale was approximately 10 min.

### 3.2. Phase II: Analysis of the Psychometric Properties of the Researcher AI Addiction Scale

#### 3.2.1. Characteristics of the Sample for Factor Analysis

The sample characteristics in Table 1 provide a demographic and professional overview of the participants involved in the factor analysis. The total sample (*n* = 718) had a mean age of 33.39 years (SD = 5.469) and an average of 11.18 years of research experience. Females comprised the majority of the sample (82.6%). Most participants were married (79.5%) and held a PhD (86.8%). The majority (84.1%) were affiliated with universities, with a smaller representation from research institutes (10%) and other affiliations (5.84%). Approximately 72.3% had access to research support, and 61.4% had received AI training.

**Table 1:** Characteristics of the sample for factor analysis.

**Variables**	**Total sample (*n* = 718)**	**Subsample 1 (*n* = 359)**	**Subsample 2 (*n* = 359)**
	**Mean (SD)**	**Mean (SD)**	**Mean (SD)**

Age (years)	33.39 (5.469)	33.5 (5.349)	33.27 (5.592)
Research experience	11.18 (3.135)	11.28 (2.965)	11.08 (3.298)
Number of published works	1.06 (1.056)	1.01 (1.083)	1.11 (1.03)

	**No. (%)**	**No. (%)**	**No. (%)**

Sex			
Female	593 (82.6)	303 (84.4)	290 (80.8)
Male	125 (17.4)	56 (15.6)	69 (19.2)
Marital status			
Not married	147 (20.5)	77 (21.4)	70 (19.5)
Married	571 (79.5)	282 (78.6)	289 (80.5)
Qualification			
Master’s degree	95 (13.2)	54 (15)	41 (11.4)
PhD.	623 (86.8)	305 (85)	318 (88.6)
Affiliation			
University	604 (84.1)	229 (83.3)	305 (85)
Research institute	72 (10)	36 (10)	36 (10)
Others	42 (5.84)	24 (6.7)	18 (5)
Access to research support			
Yes	519 (72.3)	257 (71.4)	262 (73)
No	199 (72.3)	102 (28.4)	97 (27)
Received training on AI			
Yes	441 (61.4)	215 (59.9)	226 (63)
No	277 (38.6)	144 (40.1)	133 (37)

#### 3.2.2. Factor Analysis

The absolute values of skewness and kurtosis for each subsample were used to evaluate the normality of the data. In the two subsamples, the values fell between 0.092 and 0.481 for skewness and between 0.005 and 1.195 for kurtosis. These values indicated that the data met the assumption of normality and were acceptable for factor analysis.

##### 3.2.2.1. Exploratory Factor Analysis With Subsample 1

The results of the EFA demonstrated that the KMO measure of sampling adequacy was 0.863 and the Bartlett’s test of sphericity was significant (*χ* ^2^ = 7455.843, df = 231,000, and *p* < 0.001), confirming that the data of subsample 1 were suitable for factor analysis.

**Figure 1:**
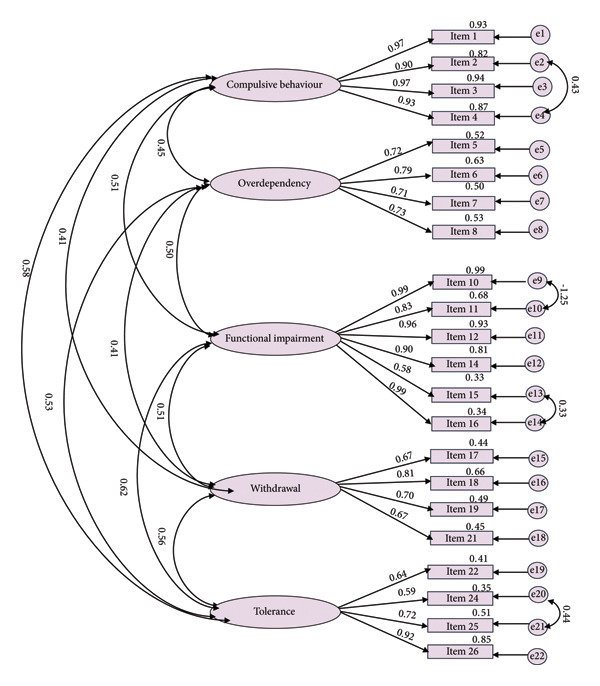
First‐order confirmatory factor analysis for the AI addiction scale. Model fit parameters: *χ* ^2^/DF = 2.287, root mean square residual (RMR) = 0.061, goodness‐of‐fit index (GFI) = 0.902, comparative fit index (CFI) = 0.962, incremental fit index (IFI) = 0.962, root mean square error of approximation (RMSEA) = 0.06, model chi‐square = 445.992, DF = 195, and *p* < 0.001. Note: All correlations between the latent variables are significant at the *p* < 0.01 level.

The 24 items of the preliminary scale were included in the EFA, and the initial analysis identified two items (items #13 and #28) with low loadings (< 0.3) across all the factors. These items were removed from the analysis. The EFA results identified five factors with eigenvalues greater than 1, collectively explaining 73.66% of the variance, as presented in Table 1s (Supporting Information). In addition to the eigenvalue‐greater‐than‐one criterion, a scree plot (Stone test) was utilized to determine the optimal number of factors to retain. The scree plot (Figure 1s, Supporting Information) showed a distinct break after the fifth factor, further supporting the selection of five factors as both the most appropriate and theoretically meaningful solution.

Most items demonstrated strong loadings (ranging from 0.596 to 0.939) on their respective factors. The communalities of the items ranged between 0.469 and 0.972, which are considered acceptable to ideal. There were no cross‐loadings among the items. These results support the validity of the remaining 22‐item scale (see Table 2). Each of the five factors was named as follows: functional impairment for Factor 1 (6 items), compulsive behavior for Factor 2 (4 items), tolerance for Factor 3 (4 items), overdependency for Factor 4 (4 items), and withdrawal for Factor 5 (4 items).

**Table 2:** Communalities and item factor loadings for exploratory factor analysis of the researchers’ AI addiction scale with Subsample 1 (*n* = 359).

	Factor					Mean	SD	*h* ^2^
	1	2	3	4	5			
ITEM 12	**0.939**	0.312	0.258	0.304	0.334	3.49	0.951	0.972
ITEM 10	**0.925**	0.324	0.258	0.316	0.344	3.48	0.957	0.961
ITEM 14	**0.834**	0.328	0.267	0.394	0.381	3.50	0.963	0.875
ITEM 11	**0.819**	0.293	0.211	0.364	0.398	3.60	0.906	0.677
ITEM 16	**0.666**	0.266	0.220	0.315	0.424	3.44	0.972	0.517
ITEM 15	**0.614**	0.180	0.314	0.324	0.376	3.34	1.109	0.469
ITEM 4	0.323	**0.920**	0.156	0.407	0.122	3.42	1.056	0.903
IITEM 1	0.315	**0.914**	0.151	0.376	0.216	3.43	1.073	0.891
ITEM 3	0.315	**0.902**	0.108	0.422	0.365	3.37	1.080	0.878
ITEM 2	0.316	**0.892**	0.213	0.350	0.205	3.46	1.074	0.803
ITEM 24	0.250	0.102	**0.853**	0.260	0.303	3.19	1.050	0.731
ITEM 22	0.255	0.186	**0.798**	0.263	0.338	3.20	1.112	0.641
ITEM 25	0.189	0.120	**0.756**	0.213	0.297	3.24	1.040	0.573
ITEM 26	0.236	0.141	**0.612**	0.184	0.269	2.67	0.726	0.551
ITEM 6	0.198	0.282	0.177	**0.794**	0.320	3.48	0.939	0.648
ITEM 5	0.305	0.303	0.282	**0.756**	0.301	3.47	0.883	0.577
ITEM 8	0.345	0.310	0.179	**0.735**	0.301	3.32	0.998	0.588
ITEM 7	0.325	0.319	0.279	**0.727**	0.372	3.49	1.008	0.538
ITEM 18	0.125	0.216	0.241	0.236	**0.812**	3.34	0.984	0.780
ITEM 17	0.285	0.396	0.228	0.129	**0.801**	3.17	1.035	0.709
ITEM 21	0.304	0.352	0.192	0.321	**0.762**	3.23	1.047	0.596
ITEM 19	0.347	0.349	0.345	0.304	**0.596**	3.25	1.107	0.577

Eigenvalue	8.059	2.855	2.354	1.721	1.216			
Percentage of the variance	36.632	12.978	10.702	7.821	5.526			

*Note:* *h* ^2^ = item communalities. Extraction method: principal axis factoring. Rotation method: Promax with Kaiser normalization. The items highlighted in bold represent the items associated with this factor.

##### 3.2.2.2. Confirmatory Factor Analysis With Subsample 2

**Figure 2:**
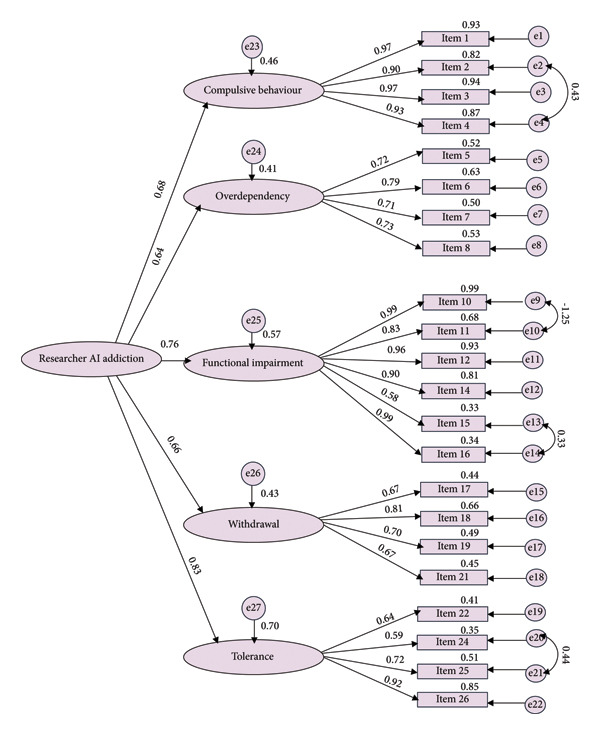
Second‐order confirmatory factor analysis for the AI addiction scale. Model fit parameters: *χ* ^2^/DF = 2.243, root mean square residual (RMR) = 0.062, goodness‐of‐fit index (GFI) = 0.903, comparative fit index (CFI) = 0.962, incremental fit index (IFI) = 0.962, root mean square error of approximation (RMSEA) = 0.059, model chi‐square = 448.532, DF = 200, and *p* < 0.001.

Figure 1 presents the results of the first‐order confirmatory factor analysis (CFA) for the Researcher AI Addiction Scale, with 22 items organized under the five factors identified from the EFA. The model fit indices for the first‐order CFA were *χ* ^2^/DF = 2.289, RMR = 0.061, GFI = 0.902, CFI = 0.962, IFI = 0.962, and RMSEA = 0.06, suggesting a good fit when accounting for model complexity and supporting the adequacy of the five‐factor model in representing the data. Figure 2 displays the results of the second‐order CFA, which evaluates the multidimensional structure of the Researcher AI Addiction Scale by treating “Researcher AI Addiction” as a second‐order latent construct encompassing the five first‐order factors. The model fit indices for the second‐order CFA were *χ* ^2^/DF = 2.243, RMR = 0.062, GFI = 0.903, CFI = 0.962, IFI = 0.962, RMSEA = 0.059, model *χ* ^2^ = 448.532, DF = 200, and *p* < 0.001, indicating an acceptable fit and confirming the multidimensionality of the scale. All 22 items were significantly loaded onto their respective first‐order factors, with standardized loadings ranging from 0.58 to 0.99. For the second‐order latent factor, the five standardized second‐order factor loadings were statistically significant, ranging from 0.64 to 0.83. Additionally, the correlations among the latent factors ranged from 0.41 to 0.62, indicating moderate associations between the constructs and supporting the multidimensionality of the Researcher AI Addiction Scale.

#### 3.2.3. Reliability and Validity Analysis

Table 3 provides evidence for the composite reliability (CR) and convergent validity of the Researchers’ AI Addiction Scale in Subsample 2. The CR values for each factor are strong, ranging from 0.809 to 0.924, exceeding the recommended threshold of 0.70 and demonstrating high internal consistency for the constructs. The AVE values range from 0.517 to 0.710, with most factors surpassing the recommended minimum of 0.50 [53], which supports adequate convergent validity. Although the AVE values for “Tolerance” (0.517), “Withdrawal” (0.547), and “Overdependency” (0.549) are close to the threshold, they still meet the criteria for sufficient convergent validity. Furthermore, the maximum shared variance (MSV) for each factor is lower than the respective AVE, reinforcing the distinctiveness of each factor. The standardized factor loadings, ranging from 0.560 to 0.999, confirm that all the items load significantly onto their respective factors, providing additional support for the scale’s structural integrity.

**Table 3:** Composite reliability and convergent validity of the researchers’ AI addiction scale using Subsample 2

Factor	Item	B	CR	AVE	MSV
Compulsive behavior	Item 1	0.965	0.970	0.889	0.335
	Item 2	0.904			
	Item 3	0.970			
	Item 4	0.931			

Overdependency	Item 5	0.722	0.827	0.545	0.279
	Item 6	0.792			
	Item 7	0.709			
	Item 8	0.727			

Functional impairment	Item 10	0.994	0.925	0.681	0.386
	Item 11	0.827			
	Item 12	0.965			
	Item 14	0.898			
	Item 15	0.579			
	Item 16	0.586			

Withdrawal	Item 17	0.667	0.805	0.510	0.319
	Item 18	0.813			
	Item 19	0.698			
	Item 21	0.668			

Tolerance	Item 22	0.644	0.814	0.530	0.386
	Item 24	0.590			
	Item 25	0.715			
	Item 26	0.920			

*Note:* B: standardized regression weights.

Abbreviations: AVE, average variance extracted; CR, composite reliability; MSV, maximum shared variance.

Table 4 shows that the square root of the AVE is higher than intervariable correlation; thus, we can conclude that our factors have good discriminant validity. Table 5 presents the reliability analysis of the scale based on the total sample (*n* = 718). The overall internal consistency of the 22‐item scale was excellent, as evidenced by Cronbach’s alpha (*α* = 0.924), McDonald’s omega (*ω* = 0.870), and the Spearman–Brown split‐half coefficient (0.814). These convergent indices collectively indicate a high degree of homogeneity among the items and confirm the stability of the scale’s measurement properties. Across the five subscales, reliability coefficients also demonstrated satisfactory to excellent internal consistency, with all values exceeding the recommended minimum threshold of 0.70. Specifically, the compulsive behavior subscale exhibited strong reliability (*α* = 0.967, *ω* = 0.835, and Spearman–Brown = 0.824), followed by overdependency (*α* = 0.958, *ω* = 0.960 and Spearman–Brown = 0.969), functional impairment (*α* = 0.925, ω = 0.896, and Spearman–Brown = 0.902), withdrawal (*α* = 0.756, *ω* = 0.757, and Spearman–Brown = 0.747), and tolerance (*α* = 0.792, *ω* = 0.802, and Spearman–Brown = 0.814).

**Table 4:** Discriminant validity of the researchers’ AI addiction scale for Subsample 2 (*n* = 368).

	Compulsive behavior	Overdependency	Tolerance	Withdrawal	Functional impairment
Compulsive behavior	**0.943**				
Over dependency	0.447	**0.738**			
Tolerance	0.579	0.528	**0.728**		
Withdrawal	0.408	0.415	0.565	**0.714**	
Functional impairment	0.514	0.497	0.621	0.514	**0.825**

*Note:* The values in the diagonal bold are the square roots of the average variance extracted, and the other values are intervariable correlations.

**Table 5:** Reliability analysis of the researchers’ AI addiction scale using the total sample (*n* = 718).

Factor	Item	Corrected item–total correlation	Cronbach alpha if item deleted	Cronbach alpha coefficient	McDonald’s omega (*ω*)	Spearman–Brown coefficient
Compulsive behavior	Item 1	0.928	0.954	0.967	0.835	0.824
	Item 2	0.894	0.964			
	Item 3	0.921	0.956			
	Item 4	0.932	0.953			

Overdependency	Item 5	0.949	0.928	0.958	0.960	0.969
	Item 6	0.782	0.976			
	Item 7	0.950	0.928			
	Item 8	0.908	0.941			

Functional impairment	Item 10	0.902	0.892	0.925	0.896	0.902
	Item 11	0.782	0.908			
	Item 12	0.903	0.892			
	Item 14	0.854	0.898			
	Item 15	0.619	0.933			
	Item 16	0.646	0.926			

Withdrawal	Item 17	0.419	0.767	0.756	0.757	0.747
	Item 18	0.657	0.639			
	Item 19	0.582	0.676			
	Item 21	0.554	0.693			

Tolerance	Item 22	0.537	0.774	0.792	0.802	0.814
	Item 24	0.651	0.715			
	Item 25	0.680	0.699			
	Item 26	0.555	0.766			

All scale	—	—	—	0.924	0.870	0.814

#### 3.2.4. Percentile‐Based Classification of RAIAS

An exploratory percentile‐based approach was used to categorize participants according to their risk of AI addiction based on RAIAS mean scores. The mean score, ranging from 1 to 5, was calculated by dividing the sum of the obtained scores by the number of items. This approach leveraged the observed score distribution to define three distinct risk tiers. Specifically, scores below the 25th percentile (*M* < 2.2) were classified as Low Risk. Scores falling between the 25th and 75th percentiles (2.2 ≤ *M* < 3.4) constituted the Moderate Risk category. Participants whose scores fell at or above the 75th percentile (*M* ≥ 3.4) were classified as High Risk, thereby segmenting the top quartile of respondents for focused analysis of problematic AI engagement.

#### 3.2.5. Controlling Common Method Variance (CMV)

To address potential CMV beyond the safeguards implemented during data collection, Harman’s single‐factor test was conducted post hoc. The results indicated that CMV was not a significant threat to the data integrity, as a single, unrotated factor accounted for only 36.621% of the total variance, which is below the recommended threshold of 50% [55].

## 4. Discussion

This study sought to respond to a critical and timely concern in academic life: the growing psychological and behavioral entanglement between researchers and AI tools. Through the development and validation of the RAIAS, our findings highlight a clear pattern of dependency behaviors across five interconnected dimensions. While these results contribute to the emerging discourse on AI overuse, they also place us at the center of a contentious academic debate—one that challenges not only how we define “addiction” in the digital age but also how we measure it.

Importantly, the RAIAS is conceptualized as an assessment rather than a diagnostic tool. Its primary purpose is to evaluate the frequency and intensity of AI‐related behaviors that may reflect problematic use, rather than to diagnose a clinical condition. The percentile‐based thresholds derived in this study (low < 25th percentile, moderate 25th–75th, and high ≥ 75th) provide a practical framework for interpreting scores. A higher total score does not indicate clinical addiction but rather signals increasing behavioral risk that warrants self‐reflection or organizational attention. Thus, the RAIAS should be used by researchers, academic leaders, and mental health educators as a screening and awareness tool to identify patterns of excessive AI reliance and to guide preventive interventions, not as a diagnostic criterion for pathological behavior.

Supporters of conceptualizing AI overuse as a behavioral addiction argue that modern technologies, especially those embedded with adaptive feedback and predictive modeling, exert an unprecedented influence on users’ cognitive processes. The findings from our study strongly align with this view. For example, the prominence of compulsive behavior and tolerance in our data suggests that researchers do not merely use AI tools frequently—they engage with them in a patterned and escalating manner that mirrors behavioral dependencies [14, 56]. The high factor loadings across these dimensions confirm that such use is not random or incidental but structured and psychologically anchored. This supports previous claims that AI’s interactivity, responsiveness, and perceived authority over cognitive tasks make it uniquely addictive compared to older forms of digital tools [12].

Yet, these findings must be weighed against a growing body of critical literature that questions the validity of labeling high engagement as pathological. Scholars such as Ciudad‐Fernández et al. [16] and Billieux et al. [15, 17] warn against over pathologizing productive or even intense interactions with technology, especially in professional settings. From this perspective, what our scale identifies as addiction may, in some cases, reflect adaptive strategies used by researchers to cope with rising performance demands, increased publication pressure, or time scarcity. Indeed, high engagement with AI tools may not necessarily indicate dysfunction, but rather a rational response to a shifting academic environment. This view is particularly salient in disciplines like nursing, where evidence generation is time‐sensitive, labor‐intensive, and directly tied to practice and policy.

However, our study does not treat frequent AI use as inherently problematic. Instead, the RAIAS distinguishes how AI is used and to what effect. For example, withdrawal symptoms—frequently debated in digital addiction studies—were not the most dominant factor in our model. Instead, it was the interplay between compulsivity, functional impairment, and emotional overdependence that marked problematic usage. Participants did not simply “miss” AI tools when unavailable; they reported decreased confidence, disrupted workflow, and loss of focus. This nuance supports the scale’s strength in capturing not just surface behaviors but deeper psychological reliance, a distinction many general digital addiction scales fail to make [11, 57].

Moreover, our findings contribute to a more refined understanding of functional impairment, one that moves beyond productivity loss to address qualitative shifts in thinking. While previous scholars have suggested that AI overuse may lead to lower‐quality work [26, 58], our data suggest a subtler erosion of methodological depth, critical engagement, and research authenticity. The strong association between tolerance and functional impairment in our model supports the hypothesis that escalating AI use may encourage a reliance on automation over reflection—an effect with long‐term implications for scientific rigor.

Nonetheless, skepticism around AI addiction remains warranted and valuable. Critics emphasize that addiction models risk individualizing what may be structural issues—such as the overwork, hyperproductivity, and precarity that characterize academic labor [20, 59]. In this light, what we interpret as problematic overuse may be a symptom of deeper systemic pressures. Our data cannot fully disentangle these factors, but the RAIAS offers a tool to begin exploring these questions empirically, with attention to the subjective experiences of researchers themselves.

The strength of the RAIAS lies in its ability to differentiate functional, intentional AI use from overuse that compromises academic judgment and independence. Unlike broader internet or smartphone addiction scales, which conflate entertainment and communication behaviors, our instrument isolates AI‐specific dependencies rooted in cognitive offloading, emotional reliance, and behavioral escalation. The results provide evidence for the validity of this distinction, especially within high‐demand, knowledge‐driven professions like nursing research.

Future research should further examine the contextual moderators of AI addiction—such as academic workload, AI literacy, digital norms in departments, and institutional expectations. Longitudinal designs are particularly needed to determine whether AI addiction develops over time or reflects an existing predisposition. Moreover, caution is needed when interpreting high scores as definitive markers of addiction rather than signals of emerging risk. Future validation studies are encouraged to refine the percentile thresholds and examine their predictive associations with external behavioral or psychological outcomes.

In sum, this study contributes to the growing literature that takes a measured and evidence‐based approach to understanding the behavioral impacts of AI on research professionals. The RAIAS offers both researchers and institutions a valuable lens to assess and reflect on AI tool usage—identifying when engagement becomes counterproductive or psychologically costly. As AI becomes further embedded in the academic landscape, tools like the RAIAS can guide balanced, ethical, and mindful integration—ensuring that technology serves research, rather than reshapes it.

### 4.1. Implications of the Study

The findings of this study offer important implications for both academic practice and institutional policy. The validated RAIAS provides a reliable tool for identifying problematic AI use patterns among researchers, enabling early detection and targeted interventions. Academic institutions can utilize the scale to promote responsible AI engagement, integrate digital well‐being initiatives into faculty development, and foster a culture of mindful technology use. For nursing researchers in particular, the RAIAS underscores the need to balance AI‐driven efficiency with the preservation of critical thinking, methodological rigor, and research integrity—skills essential for advancing evidence‐based practice and professional growth in the AI era.

### 4.2. Strengths and Limitations

This study has several strengths. The RAIAS was developed to address the growing prominence of AI tool misuse, an emerging challenge in the realms of research and professional practice. Methodologically, it followed a rigorous process encompassing a comprehensive literature review, expert validation, pilot testing, and psychometric evaluation, ensuring both content validity and structural validity. The robustness of the scale is evidenced by strong psychometric properties, including high internal consistency and robust factor loadings confirmed through EFA and CFA. Moreover, the scale was validated using a large sample of researchers from diverse universities, ensuring the generalizability of the findings.

However, this study has some limitations. While the RAIAS was deliberately constructed as a general researcher instrument and its theoretical basis is domain‐agnostic, the psychometric evaluation reported here used a sample composed primarily of nursing researchers in Egypt and Saudi Arabia. Therefore, although the scale shows strong psychometric properties in this sample, further research is required to confirm cross‐disciplinary generalizability. Future studies should examine measurement invariance across research fields (e.g., clinical, social sciences, and STEM) and across cultural contexts to ensure the RAIAS measures the same constructs equivalently among different researcher populations.

## 5. Conclusion

The researchers’ AI addiction scale is a valid and reliable tool for assessing AI addiction in research contexts. With five dimensions—compulsive behavior, overdependency, functional impairment, withdrawal, and tolerance—the scale captures the multifaceted nature of AI dependency. Strong psychometric results, including high internal consistency, convergent validity, and structural integrity, affirm its robustness. Tailored to the professional use of AI tools, the scale fills a critical gap in addiction research, offering insights into balanced AI integration while preserving essential research skills.

## Ethics Statement

The study protocol was approved by the Research Ethics Committee of the Faculty of Nursing at Alexandria University, Egypt (IRB00013620 AU:20‐8‐419). Before participating, all the respondents provided electronic informed consent, detailing the study’s purpose, voluntary nature, confidentiality of responses, and participants’ right to withdraw at any time.

## Disclosure

All authors of this paper have read and approved the final version submitted.

## Conflicts of Interest

The authors declare no conflicts of interest.

## Author Contributions

Ahmed Abdelwahab Ibrahim El‐Sayed: writing–original draft, review, and editing, visualization, validation, data curation, investigation, resources, methodology, supervision, and conceptualization. Samira Ahmed Alsenany: writing–original draft, review, and editing, validation, resources, and conceptualization. Maha Gamal Ramadan Asal: writing–original draft, review, and editing, validation, methodology, investigation, formal analysis, supervision, and conceptualization. Ibrahim Alasqah: writing–original draft, review, and editing, visualization, validation, data curation, investigation, resources, and methodology.

## Funding

The authors would like to thank the Deanship of Graduate Studies and Scientific Research at Qassim University, Saudi Arabia, for financial support (QU‐APC‐2025).

## Supporting Information

This manuscript contains 1 supporting information that shows domains and items of the AI addiction scale as well as Total Variance Explained by the Exploratory Factor Analysis.

## Supporting information


**Supporting Information** Additional supporting information can be found online in the Supporting Information section.

## Data Availability

The data that support the findings of this study are available from the corresponding author upon reasonable request.
