# Supplementary material for: Development and Validation of Artificial Intelligence Addiction Scale for Researchers: A Methodological Study
Source: J Nurs Manag. 2025 Dec 18;2025:8458533. doi: 10.1155/jonm/8458533 (PMC12714078; doi:10.1155/jonm/8458533)
Supplement: Supplementary file 1 — Supporting Information Additional supporting information can be found online in the Supporting Information section. [file JONM-2025-8458533-s001.docx]

**Development and Validation of Researchers’ AI Addiction Scale: A Methodological Study**

**Preliminary items for researchers’ AI Addiction Scale**

**Compulsive Behavior**

1. I use AI tools more than I initially intended.
2. I think about using AI tools even when I am not currently using them.
3. I feel compelled to use AI tools even when I should focus on other tasks.
4. I have tried to cut down my use of AI tools but failed.

**Over Dependency**

1. I depend on AI tools to generate ideas or solve research problems.
2. I rely on AI tools to make decisions without careful evaluation.
3. I use AI tools even for simple tasks that I could easily do myself.
4. I use AI tools to avoid learning complex research topics on my own.
5. I find myself planning the next time I can use AI tools. **(R)**

**Functional Impairment**

1. My reliance on AI tools reduces my ability to develop critical research skills.
2. I find it hard to start or finish research tasks without AI tools.
3. My research quality has declined due to over-reliance on AI tools.
4. I use AI tools to shortcut the research process. **(R)**
5. Using AI tools distracts me from my research goals and deadlines.
6. My use of AI tools has negatively affected my relationships with colleagues or supervisors.
7. Using AI tools in research reduces my time for personal and work responsibilities.

**Withdrawal**

1. I feel anxious when I cannot access or use AI tools for my research tasks.
2. I feel a strong urge to return to using AI tools when I am unable to access them.
3. I find it difficult to concentrate on my research without using AI tools.
4. I feel a sense of emptiness when I am not using AI tools. **(R)**
5. I become frustrated or irritable when I cannot use AI tools for my research.

**Tolerance**

1. I need to use AI tools repeatedly for a long time to get the same satisfaction in research.
2. I feel that AI tools are less effective than when I first started using them. **(R)**
3. I rely more on AI tools and use them more to get similar results.
4. I find myself using AI tools in situations where I previously did not need them.
5. I feel the need to explore new features of AI tools to maintain my interest.
6. My tolerance for using other research tools has decreased because of AI tools. **(R)**
7. I constantly look for new ways to integrate AI tools into my research. **(R)**

**(R): items removed from the scale**

**Table 1s: Total Variance Explained by the Exploratory Factor Analysis (EFA)**

| **Total Variance Explained** | | | | | | | |
| --- | --- | --- | --- | --- | --- | --- | --- |
| Factor | Initial Eigenvalues | | | Extraction Sums of Squared Loadings | | | Rotation Sums of Squared Loadings^a^ |
|  | Total | % of Variance | Cumulative % | Total | % of Variance | Cumulative % | Total |
| 1 | 8.059 | 36.632 | 36.632 | 7.777 | 35.349 | 35.349 | 5.807 |
| 2 | 2.855 | 12.978 | 49.611 | 2.672 | 12.144 | 47.494 | 5.203 |
| 3 | 2.354 | 10.702 | 60.313 | 2.039 | 9.268 | 56.761 | 3.332 |
| 4 | 1.721 | 7.821 | 68.134 | 1.352 | 6.146 | 62.907 | 4.402 |
| 5 | 1.216 | 5.526 | 73.660 | .865 | 3.932 | 66.840 | 5.259 |
| 6 | .799 | 3.630 | 77.290 |  |  |  |  |
| 7 | .729 | 3.314 | 80.604 |  |  |  |  |
| 8 | .638 | 2.899 | 83.503 |  |  |  |  |
| 9 | .545 | 2.476 | 85.979 |  |  |  |  |
| 10 | .512 | 2.329 | 88.307 |  |  |  |  |
| 11 | .424 | 1.926 | 90.233 |  |  |  |  |
| 12 | .401 | 1.824 | 92.057 |  |  |  |  |
| 13 | .350 | 1.589 | 93.646 |  |  |  |  |
| 14 | .274 | 1.244 | 94.890 |  |  |  |  |
| 15 | .247 | 1.122 | 96.012 |  |  |  |  |
| 16 | .236 | 1.071 | 97.083 |  |  |  |  |
| 17 | .222 | 1.009 | 98.092 |  |  |  |  |
| 18 | .167 | .760 | 98.852 |  |  |  |  |
| 19 | .112 | .511 | 99.363 |  |  |  |  |
| 20 | .071 | .322 | 99.685 |  |  |  |  |
| 21 | .067 | .303 | 99.988 |  |  |  |  |
| 22 | .003 | .012 | 100.000 |  |  |  |  |
| Extraction Method: Principal Axis Factoring. | | | | | | | |
| a. When factors are correlated, sums of squared loadings cannot be added to obtain a total variance. | | | | | | | |


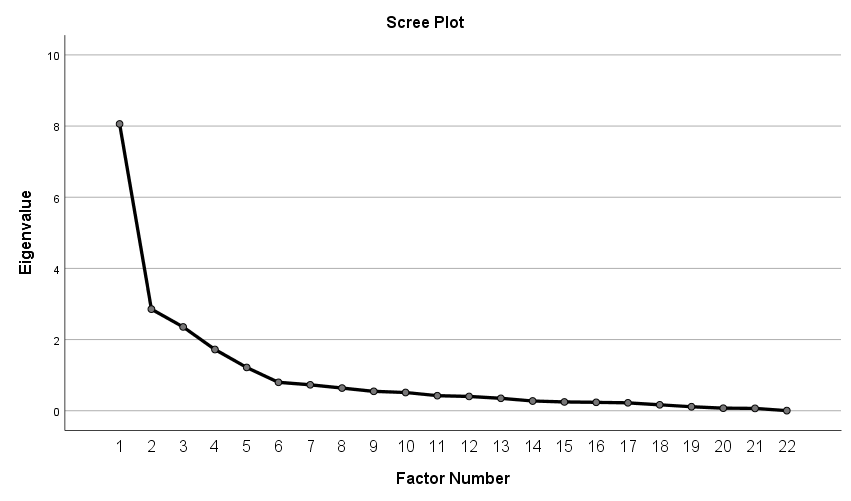


**Figure 1s: Scree Plot for Factor Retention in the Exploratory Factor Analysis**
